# Prevention of Treacher Collins syndrome craniofacial anomalies in mouse models via maternal antioxidant supplementation

**DOI:** 10.1038/ncomms10328

**Published:** 2016-01-21

**Authors:** Daisuke Sakai, Jill Dixon, Annita Achilleos, Michael Dixon, Paul A. Trainor

**Affiliations:** 1 Organization for Research Initiatives and Development, Doshisha University, Karasuma Higashi-iru, Imadegawa-dori, Kamigyo, Kyoto 602-8580, Japan; 2 Dental School, Faculty of Medical and Human Sciences, Manchester Academic Health Sciences Centre, Michael Smith Building, University of Manchester, Oxford Road, Manchester M13 9PT, UK; 3 Stowers Institute for Medical Research, 1000 E. 50th Street, Kansas City, Missouri 64110, USA; 4 Faculty of Life Sciences, Michael Smith Building, University of Manchester, Oxford Road, Manchester M13 9PT, UK; 5 Department of Anatomy and Cell Biology, University of Kansas Medical Center, Kansas City, Kansas 66160, USA

## Abstract

Craniofacial anomalies account for approximately one-third of all birth defects and are a significant cause of infant mortality. Since the majority of the bones, cartilage and connective tissues that comprise the head and face are derived from a multipotent migratory progenitor cell population called the neural crest, craniofacial disorders are typically attributed to defects in neural crest cell development. Treacher Collins syndrome (TCS) is a disorder of craniofacial development and although TCS arises primarily through autosomal dominant mutations in *TCOF1*, no clear genotype–phenotype correlation has been documented. Here we show that *Tcof1* haploinsufficiency results in oxidative stress-induced DNA damage and neuroepithelial cell death. Consistent with this discovery, maternal treatment with antioxidants minimizes cell death in the neuroepithelium and substantially ameliorates or prevents the pathogenesis of craniofacial anomalies in *Tcof1*^+/−^ mice. Thus maternal antioxidant dietary supplementation may provide an avenue for protection against the pathogenesis of TCS and similar neurocristopathies.

## Abstract (web summary)

The *TCOF1* gene is mutated in Treacher Collin's syndrome, a congenital craniofacial syndrome. Here, the authors show that *Tcof1* loss-of-function results in oxidative stress induced DNA damage and neuroepithelial cell death, and addition of antioxidants to pregnant mutant mice protected against these defects.

## Graphical abstract

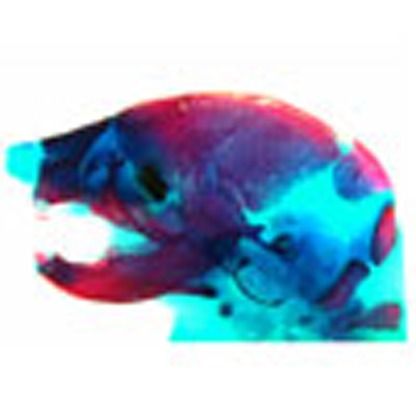

Neural crest cells comprise a multipotent, migratory progenitor cell population that generate most of the cartilage, bone and connective tissues of the head and face during embryonic development^1^,^2^. Congenital craniofacial malformations are therefore largely attributed to defects in the formation, migration, proliferation and/or differentiation of neural crest cells^3^. Development of therapeutic avenues for the prevention of congenital craniofacial anomalies depends upon a thorough appreciation of the genetic and cellular mechanisms that regulate neural crest cell development in association with the etiology and pathogenesis of individual malformation syndromes. Treacher Collins syndrome (TCS, OMIM number 154500) is a severe congenital disorder of craniofacial development and occurs with an incidence of one in 50,000 live births. The major characteristics of TCS include cleft palate, hypoplasia of the facial bones, particularly the mandible and zygomatic complex, downward slanting of the palpebral fissures and malformation of the external and middle ear^4^.

TCS is primarily associated with autosomal dominant mutations in the *TCOF1* gene which is located on chromosome 5 in humans^5^. *TCOF1* encodes a 144 kDa nucleolar phosphoprotein called Treacle, which functions in ribosomal DNA transcription via direct binding of upstream binding factor and RNA polymerase I in the nucleolus. *Tcof1* is expressed broadly throughout the embryo, with particularly strong activity in the neuroepithelium where it plays an essential role in cell survival. Analyses of a *Tcof1*^+/−^ mouse model of TCS determined that this disorder arises through extensive apoptosis of neuroepithelial cells, and a deficiency in the generation and proliferation of neural crest cells which are the precursors of the craniofacial skeleton^4^,^6^,^7^. Furthermore, *Tcof1* haploinsufficiency leads to deficient ribosome biogenesis^8^. Deficient ribosome biogenesis can cause nucleolar stress activation of p53 (ref. 9), and consistent with this mechanism, stabilization of p53 protein and activation of p53-responsive pro-apoptotic genes is observed in the neuroepithelium of *Tcof1*^+/−^ embryos^7^. Thus, deficient ribosome biogenesis has been proposed to be responsible for the p53-mediated high levels of neuroepithelial cell death observed in *Tcof1*^+/−^ embryos. Interestingly, genetic and pharmacological inhibition of p53 can suppress neuroepithelial apoptosis in *Tcof1*^+/−^ embryos and prevent the pathogenesis of craniofacial anomalies characteristic of TCS^7^. However, this rescue occurs without restoration of ribosome biogenesis, implying that *Tcof1*/Treacle may play important roles in neuroepithelial cell and neural crest cell survival in addition to, and distinct from, its currently recognized function in ribosome biogenesis.

Neural crest cells are derived from the neuroepithelium and in this study we discovered that the neuroepithelium exists endogenously in a highly oxidative state and is very sensitive to exogenous oxidative stress. More importantly we show that *Tcof1* haploinsufficiency results in oxidative stress-induced neuroepithelial cell death in association with DNA damage. *Tcof1*/Treacle associates with the MRNM complex (MRE11, Rad51, NBS1 and MDC1) and functions in DNA damage response/repair to limit oxidative stress induced, neuroepithelial cell death, during early embryogenesis. Consistent with this idea, maternal antioxidant supplementation can ameliorate the pathogenesis of craniofacial abnormalities characteristic of TCS. Thus in addition to its role in ribosome biogenesis, *Tcof1* may also be required for protection of the neuroepithelium from oxidative stress-induced cell death.

## Results

### Treacle interacts with DNA damage response proteins

**Figure 1:**
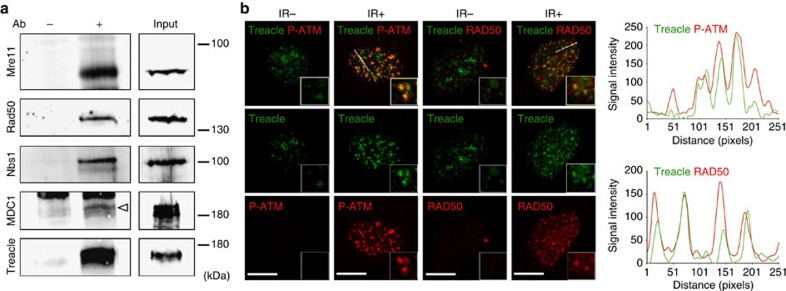
Treacle interacts with Mre11–Rad50–Nbs1–MDC1 complex and localizes to DNA damage-induced foci. (**a**) Interaction between endogenous Treacle and endogenous Mre11, Rad50, Nbs1 and MDC1 were detected by immunoprecipitation with (+) or without (−) anti-TCOF1 antibody. Proteins in the immunoprecipitated fraction were detected using antibodies to the proteins indicated on the left side. Arrowhead indicates a band of MDC1. (**b**) Immunofluorescence images of HeLa cells with (IR+) or without (IR−) X-ray irradiation. DNA damage-induced foci within nucleus were confirmed by co-immunostaining of anti-phosphorylated ATM (P-ATM), anti-RAD50 and anti-Treacle antibodies. Higher magnification images are shown as insets. Scale bar, 5 μm. Plot profiles show signal intensities along an oblique line quantified by ImageJ (five independent experiments and one representative image shown).

To explore the potential for novel *Tcof1*/Treacle functions during embryogenesis, we established HEK293-derived cell lines stably expressing FLAG-tagged Treacle, and following immunoprecipitation with an anti-FLAG antibody, identified proteins that interacted with Treacle using multidimensional protein identification technology (MudPIT)^10^,^11^. Many proteins were identified including casein kinase 2, subunits of RNA polymerase I and III, and upstream binding factor, which have previously been demonstrated to associate with Treacle^8^,^12^ validating the success of our proteomics approach. Surprisingly, we discovered that Treacle interacts with the MRNM complex which plays critical roles in the DNA damage response (Supplementary Fig. 1a). The MRN complex functions as a DNA damage sensor by recognizing and binding to the broken ends of DNA^13^. The MRN complex also participates in activating the checkpoint kinase ataxia telangiectasia mutated (ATM) in response to DNA damage and together with MDC1 reinforces and enhances ATM activity^14^,^15^,^16^. The interaction of the endogenous MRNM complex with FLAG-Treacle was confirmed by immunoprecipitation followed by western blot. Endogenous Mre11, Rad50, Nbs1 and MDC1 were each precipitated with anti-FLAG antibody from FLAG-Treacle-expressing cells (Supplementary Fig. 1b). We further verified the interaction between endogenous TCOF1 and endogenous MRNM complex proteins by immunoprecipitation using anti-TCOF1 antibody. Endogenous MRNM proteins were also co-precipitated with endogenous TCOF1 from non-irradiated HEK293 cells (Fig. 1a). These observations suggested that Treacle may play a novel role in the DNA damage response and repair process in association with the MRNM complex.

### Treacle localizes to sites of DNA damage

Therefore we hypothesized that in response to DNA damage, Treacle might localize to DNA lesions together with the MRNM complex. In control non-irradiated HeLa cells, Treacle is observed largely in nucleoli consistent with previous observations and its known functions in ribosome biogenesis^8^. Although Treacle co-precipitated with the MRNM complex in control non-irradiated cells, immunofluorescence did not reveal any specific co-localization of Treacle and RAD50 suggesting that the MRNM–Treacle complex is probably endogenously dispersed in nucleoplasm (Fig. 1b; IR−). In contrast, after X-ray irradiation we observed the appearance of numerous, widely distributed Treacle-labelled foci within individual nuclei (Fig. 1b; IR+). Interestingly, the formation of Treacle foci occurred without upregulation of its expression upon irradiation (Supplementary Fig. 2). More importantly, Treacle foci were frequently co-labelled with the key DNA damage response proteins, phosphorylated ATM at Serine 1981 (P-ATM) and Rad50, a component of MRNM complex (Fig.1b). To further substantiate our observations, we generated plot profiles and quantified signal intensities to illustrate the degree of overlap and our analysis clearly showed extensive co-localization of Treacle-labelled foci with P-ATM and Rad50-labelled foci post irradiation (Fig. 1b). Taken together, these results demonstrate that in response to irradiation-induced DNA damage, Treacle can localize to DNA lesions in association with DNA damage response proteins.

### Treacle localization with the MRN complex is MDC1 dependent

**Figure 2:**
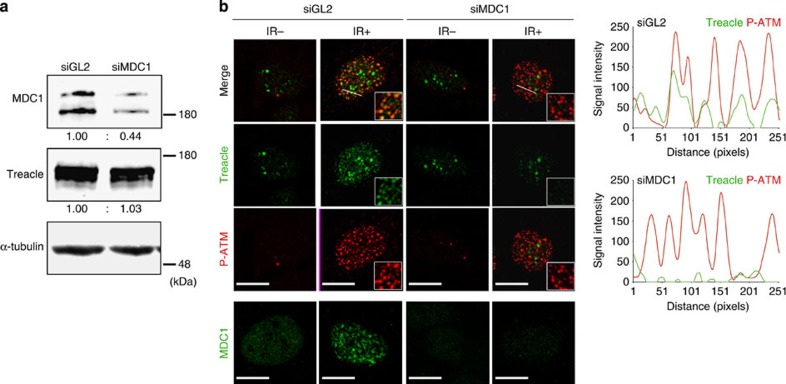
Localization of Treacle to DNA damage-induced foci depends on MDC1. (**a**) Knockdown efficacy of siRNA against MDC1 (siMDC1) was analysed by western blotting with indicated antibodies. Signals were normalized to α-tubulin signals. Fold increase of relative values are presented. (**b**) Immunofluorescence images of MDC1 knockdown cells. HeLa cells transfected with control (siGL2) and siMDC1 were immunostained with antibodies to Treacle, P-ATM and MDC1 before (IR−) or 1 h after 10 Gy irradiation (IR+). Higher magnification images are shown as insets. Scale bar, 5 μm. Plot profiles show signal intensities along an oblique line quantified by ImageJ (five independent experiments, one representative image shown).

MDC1 serves as a scaffold for the accumulation of DNA damage response proteins at DNA damage sites and also functions to amplify the DNA damage response^17^. Thus, the loss of MDC1 prevents the localization of DNA damage response/repair proteins, including the MRN complex, to sites of DNA damage^18^,^19^. Since Treacle interacts with the MRNM complex, we hypothesized that MDC1 might also be required for the localization of Treacle. We first validated the specificity of small interfering RNA (siRNA) against MDC1 (siMDC1; ref. 18). Transfection of siMDC1 significantly reduced MDC1 expression, while TCOF1 expression was not altered (Fig. 2a). Furthermore, the formation Rad50 and Nbs1 foci in response to DNA damage, was diminished by MDC1 silencing. Line plots of signal intensity clearly showed the reduction of Rad50 and Nbs1 foci in MDC1 knockdown cells, proving the efficiency and specificity of siMDC1 (Supplementary Fig. 3). We next examined the requirement for MDC1 in the localization of Treacle at sites of DNA damage. Following MDC1 knockdown, Treacle did not localize at foci in response to DNA damage (Fig. 2b). The diminishment of Treacle foci in MDC1 knockdown cells was further verified by quantitative analysis of signal intensity plot profiles (Fig. 2b). In contrast, the localization of P-ATM which is an upstream effecter of MDC1 in response to DNA damage, was not altered (Fig. 2b). These results demonstrate that Treacle can localize to sites of DNA damage together with the MRN complex in an MDC1-dependent manner. Thus *TCOF1* may play an important role in the DNA damage response/repair process.

### *Tcof1* loss-of-function is associated with DNA damage *in vivo*

**Figure 3:**
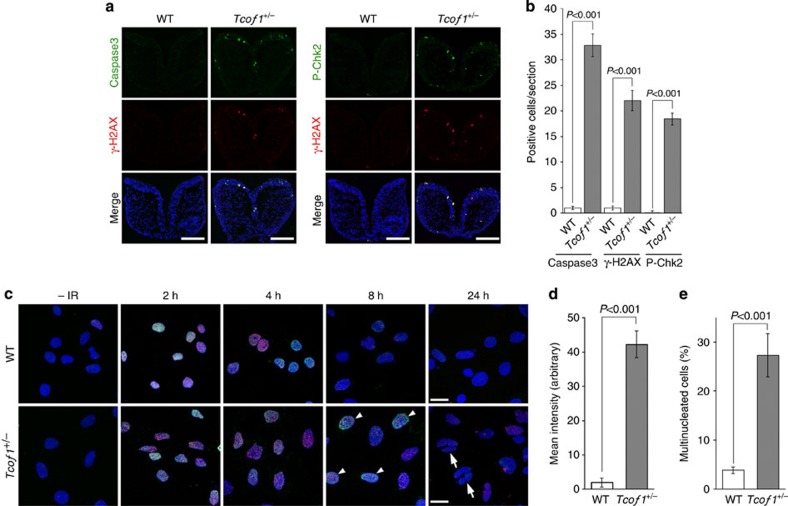
*Tcof1* deficiency causes dysfunction of DNA damage repair and subsequent apoptosis. (**a**) γ-H2AX-, phosphorylated Chk2 at T68 (P-Chk2)- and cleaved Caspase 3 (Caspase 3)-positive cells were detected by immunofluorescence at fore–mid brain level of E8.5 embryos (somite number 6–8). Scale bar, 100 μm. (**b**) The number of Caspase 3- and γ-H2AX-positive cells within the neuroepithelium was counted in 20 histological sections obtained from five embryos. Data are mean±s.e. Statistical differences were assessed with Student's *t*-test, and *P* values are shown. (**c**) Accumulation of P-ATM (red) and γ-H2AX (green) in MEF cells derived from wild-type (WT) and *Tcof1*^+/−^ embryos were analysed by immunostaining after indicating time of X-ray irradiation or without irradiation (IR−). Arrowheads indicate cells having DNA damage-induced foci due to the delay in the DNA damage repair. Arrows indicate cells showing the multinucleation caused by severe DNA damage repair deficiency. Scale bars, 10 μm. (**d**) γ-H2AX and P-ATM intensity examined in culture 8 h after of irradiation was quantified with the ImageJ. Data are mean±s.e. of 10 independent cells. Statistical differences were assessed with Student's *t*-test, and *P* value is shown. (**e**) Percentages of cells showing multinucleation in culture 24 h after of irradiation are presented. Data are mean±s.e. of 50 independent cells. Statistical differences were assessed with Student's *t*-test, and *P* value is shown.

Mouse models of TCS have revealed that extensive neuroepithelial apoptosis leads to the diminished generation and proliferation of migrating neural crest cells, which underlies the pathogenesis of craniofacial anomalies observed in these mice^6^,^7^. Given the association between *Tcof1*/Treacle and the DNA damage response and repair process, we hypothesized that the neuroepithelial cell death observed in *Tcof1*^+/−^ embryos may be associated with DNA damage. The MRN complex recruits ATM to DNA strand breaks, where it functions as a primary activator of the cellular response to DNA damage. Not only does ATM autophosphorylate itself, but it also rapidly phosphorylates H2AX (then referred to as γ-H2AX) in response to DNA damage induction^20^,^21^. Western blot analysis showed that the levels of P-ATM in *Tcof1*^+/−^ embryos were increased by about 1.7- and 1.5-fold over controls, while γ-H2AX was increased by 4.8- and 2.2-fold in 6- and 8-somite stage E8.5 *Tcof1*^+/−^ embryos, respectively (Supplementary Fig. 4a,b). More importantly however, section immunostaining revealed that γ-H2AX-positive cells were specifically detected in the neuroepithelium of E8.5 *Tcof1*^+/−^ embryos in contrast to wild-type littermates and this is consistent with the origins of neural crest cells (Fig. 3a,b). Because γ-H2AX is also expressed in cells undergoing DNA fragmentation during apoptosis^22^, we confirmed the activation of the DNA damage response/repair pathway in *Tcof1*^+/−^ embryos with other standard DNA damage markers such as phosphorylated Chk2 (P-Chk2) in combination with γ-H2AX (Fig. 3a,b). Chk2 is activated via phosphorylation at threonine 68 by ATM in response to DNA damage and transmits the signal from the DNA damage response pathway to the apoptotic pathway^23^,^24^,^25^. Consistent with this process, P-Chk2-labelled cells were co-stained with γ-H2AX in *Tcof1*^+/−^ embryos, and furthermore, the γ-H2AX-positive cells were typically co-labelled with cleaved Caspase 3 antibody, a marker for apoptotic cells (Fig. 3a). Thus the previously observed neuroepithelial stabilization of p53 (ref. 7) and consequent induction of neuroepithelial apoptosis in *Tcof1*^+/−^ embryos may potentially occur via Chk2 activation. Therefore, DNA damage could indeed contribute to the neuroepithelial cell apoptosis observed in the pathogenesis of TCS. Furthermore, our results suggest that *Tcof1/*Treacle potentially plays an important role in the DNA damage response and repair process *in vivo*.

### *Tcof1* loss-of-function perturbs DNA damage repair

Consistent with these ideas, we hypothesized that *Tcof1*/Treacle loss-of-function should impair the DNA damage response/repair process *in vivo*. Consequently we irradiated mouse embryonic fibroblast (MEF) cells derived from wild-type control and *Tcof1*^+/−^ embryos. In control MEF cells derived from wild-type embryos, P-ATM- and γ-H2AX-labelled foci were observed within 2 h post-X-ray irradiation. Such foci gradually decreased in both size and number during 8 h culture, and had completely disappeared within 24 h. In contrast, X-ray irradiated *Tcof1*^+/−^ MEF cells which were haploinsufficient for *Tcof1*, exhibited P-ATM- and γ-H2AX-labelled foci that persisted in size and number for >8 h, continuing for up to 24 h of culture (Fig. 3c,d; arrowheads). These results are indicative of a delay or arrest of DNA damage repair in *Tcof1*^+/−^ MEF cells compared with wild-type control MEF cells. Moreover, multinucleation which is a byproduct of defects in DNA damage response/repair was significantly increased in *Tcof1*^+/−^ MEF cells (27.5%) compared with wild-type MEF cells (7.7%) after 24 h culture (Fig. 3c,e; arrows). These results strongly suggest that *Tcof1*/Treacle haploinsufficiency perturbs the ability to repair DNA damage.

**Figure 4:**
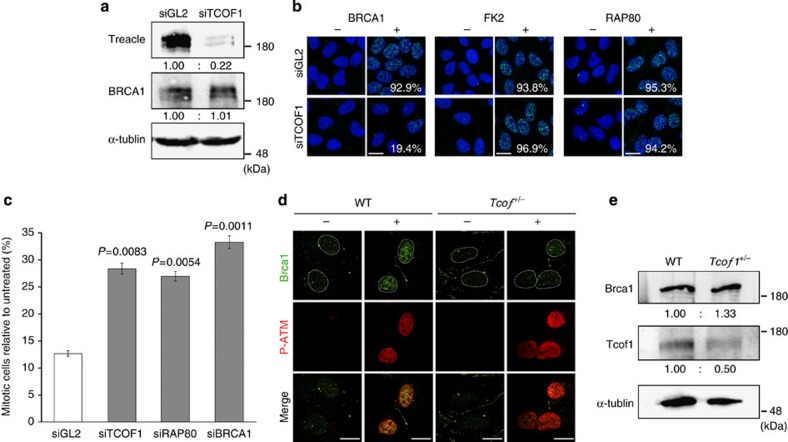
Loss of *TCOF1* leads to mislocalization of BRCA1. (**a**) Knockdown efficacy of siRNA against TCOF1 (siTCOF1) was analysed by western blotting with indicated antibodies. Signals were normalized to α-tubulin signals. Fold increase of relative values are presented. (**b**) DNA damage-induced foci formation in *Tcof1* knockdown cells was analysed by immunofluorescence using indicated antibodies. The percentage shown in each figure was calculated by counting the number of cells having more than 10 DNA damage-induced foci in 1,000 cells. Scale bar, 10 μm. (**c**) Relative increase of G2/M phase cells after 3 Gy irradiation was quantified by FACS analysis. Data are mean±s.e. from seven independent experiments. Statistical differences were assessed with Student's *t*-test, and *P* values were shown. (**d**) BRCA1 foci formation in wild-type (WT) and *Tcof1*^+/−^ MEF cells upon DNA damage (+) was analysed by immunofluorescence using P-ATM and Brca1 antibodies. Non-treated MEF cells were used as control (−). Scale bar, 10 μm. (**e**) Expression levels of Brca1 in wild-type (WT) and *Tcof1*^+/−^ MEF cells were analysed by western blotting with indicated antibodies. Signals were normalized to α-tubulin signals. Fold increase of relative values are presented.

To more precisely define the role that *TCOF1*/Treacle potentially plays in the DNA damage response/repair process, we examined the effect of X-ray irradiation in association with *TCOF1* loss-of-function on ATM, and its downstream DNA damage response proteins. In HeLa cells exposed to X-ray irradiation, knockdown of *TCOF1* using siRNAs (Supplementary Fig. 5; HSS110575, 110248 and 110249) did not affect the formation of γ-H2AX-, P-ATM-, NBS1-, RAD50-, MDC1- and 53BP1-labelled DNA damage-induced foci (Supplementary Fig. 6). This is consistent with the presence of γ-H2AX and P-ATM *in vivo* in *Tcof1*^+/−^ embryos. However, in contrast, BRCA1-labelled foci were considerably decreased after TCOF1 knockdown (Supplementary Fig. 6). In fact, following transfection of siTCOF1, >80% of the cells failed to exhibit BRCA1-labelled foci (Fig. 4b). We further confirmed that HSS110575 (siTCOF1) silencing of TCOF1 did not affect BRCA1 expression (Fig. 4a), only its localization. The localization of BRCA1 to sites of DNA damage is thought to be mediated by RAP80 which binds to ubiquitinated histones around DNA lesions^26^,^27^,^28^. FK2 (conjugated ubiquitin) and RAP80-labelled foci were formed in *TCOF1* knockdown cells post-X-ray irradiation (Fig. 4b), implying that the reduction of BRCA1 foci formation is not due to a defect in the ubiquitylation of histones or the loss or mislocalization of RAP80.

BRCA1 regulates cell cycle checkpoints and the subsequent recruitment of DNA damage repair enzymes at DNA lesions^29^. Our fluorescence-activated cell sorting and cell cycle analyses revealed that depletion of *TCOF1* impairs the G2/M checkpoint (Fig. 4c). The ratio of cells in G2/M without irradiation is 6.36% in GL2 (control), 3.60% in siTcof1, 7.18% in siRAP80 and 6.60% in siBRCA. This suggests that mitotic progression is impaired in the absence of external perturbation which is consistent with our previous findings^30^. However, the ratio of G2/M cells significantly increased after irradiation, suggesting that Tcof1 may be required for the G2/M checkpoint that is induced by DNA damage. Collectively these observations suggest that *TCOF1*/Treacle may play an important role in the DNA damage response/repair through BRCA1 recruitment to and/or maintenance at sites of DNA damage. We further examined whether DNA damage-induced Brca1-labelled foci formation was also impaired by haploinsufficiency of *Tcof1*. Indeed, Brca1-labelled DNA damage-induced foci were significantly reduced in *Tcof1*^+/−^ MEF cells, whereas in contrast they were readily detected in wild-type MEF cells upon DNA damage (Fig. 4d). The absence of BRCA1-labelled foci was not due to a reduction in *Brca1* expression caused by haploinsufficiency of *Tcof1* (Fig. 4e). Therefore these results indicate that the neuroepithelial apoptosis in *Tcof1*^+/−^ embryos may be associated with perturbed BRCA1 localization in response to DNA damage.

### The neuroepithelium exists in a highly oxidative state

**Figure 5:**
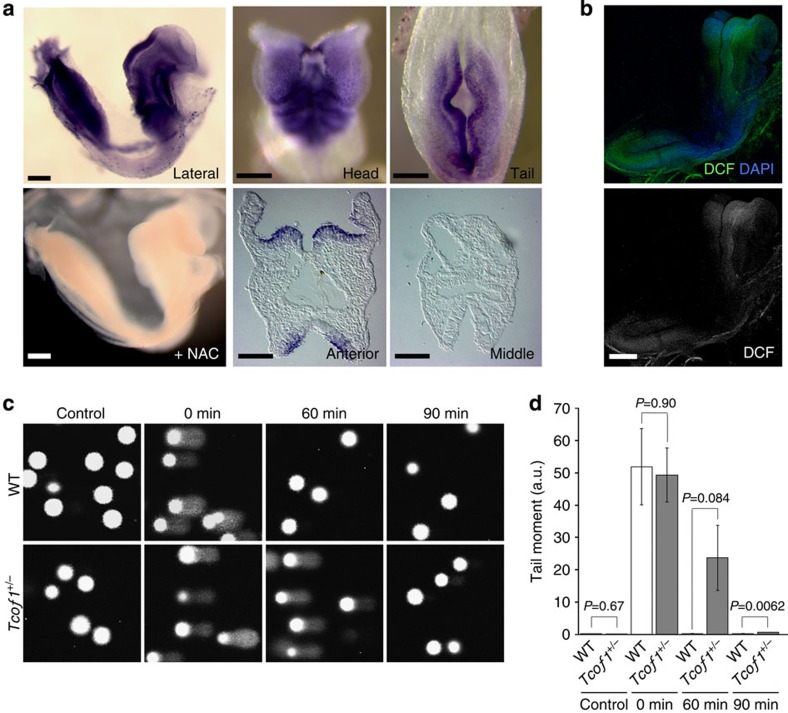
High levels of oxidation in the neuroepithelium of wild-type embryos. (**a**) Endogenous oxidation was visualized by reduction of NBT. NBT formazan is detected in anterior (head) and posterior (tail) regions of the neural plate. Sections at fore–midbrain level (anterior) show the specific staining of neuroepithelial cells. NBT staining was not detected at the lower cervical level (middle). Treatment with antioxidant, NAC prevents formation of NBT formazan, indicating specificity of NBT staining. Scale bar, 100 μm. (**b**) Endogenous ROS were visualized by incubation with fluorogenic probe, H_2_-DCFDA. Fluorescence of DCF was shown. Scale bar, 100 μm. (**c**) Time-course kinetics of DNA damage repair in wild-type (WT) and *Tcof1*^+/−^ MEF cells treated with or without H_2_O_2_ was analysed by alkaline Comet assay. (**d**) Tail moment is determined by OpenComet and presented as mean±s.e. of 50 cells from three independent experiments. Statistical differences were assessed with Student's *t*-test, and *P* values were shown.

Genotoxic factors such as ultraviolet exposure and X-ray irradiation can generate reactive oxygen species (ROS), which are known to damage DNA in a process called oxidative stress-induced DNA damage^31^,^32^,^33^,^34^. However, ROS are also generated as natural byproducts of respiration and via NADPH oxidase activity under normal physiological conditions^35^. We hypothesized that endogenously produced ROS may be associated with DNA damage and elevated levels of apoptosis in the pathogenesis of TCS. Using nitroblue tetrazolium (NBT) staining which forms an insoluble formazan in the presence of superoxide^36^, as well as a fluorogenic ROS indicator (H_2_-DCFDA), we detected high levels of ROS in E8.5 wild-type embryos (Fig. 5a,b). Transverse sections of the cranial region clearly demonstrated that neuroepithelial cells exist endogenously in a highly oxidative state relative to cells in the non-neural ectoderm, mesoderm and endoderm (Fig. 5a).

We postulated that high levels of endogenous ROS could lower the threshold for oxidative stress-induced apoptosis, such that neuroepithelial cells might be highly sensitive to excess ROS compared with other cells. Therefore to test this idea, we treated whole E8.5 wild-type mouse embryos in culture with 3-nitropropionic acid (3-NP), a strong ROS generator^37^,^38^,^39^. Even though 3-NP induced the production of ROS throughout the whole embryo (Supplementary Fig. 7c,d), exposure to 1, 2 or 4 mM 3-NP led to a substantial increase in the number of TUNEL-positive apoptotic cells specifically in the anterior neuroepithelium (Supplementary Fig. 7a,b). This observation demonstrates that neuroepithelial cells exist in an endogenously high oxidative state and also that neuroepithelial cells are particularly sensitive to additional exogenous oxidative stress. Interestingly, endogenous oxidation was not increased in *Tcof1*^+/−^ embryos compared with wild-type littermates, indicating that the neuroepithelial apoptosis observed in *Tcof1*^+/−^ embryos was not due to excessive production of ROS, but rather a diminished capacity to cope with the endogenous effects of ROS (Supplementary Fig. 8). Our data therefore demonstrate that haploinsufficiency of *Tcof1*/Treacle perturbs the DNA damage response/repair process, which in turn affects the ability of *Tcof1*^+/−^ embryos to cope with the endogenously high levels of oxidation.

In further support of this concept, we performed comet assays, firstly as a quantitative measure of DNA damage in wild-type and *Tcof1*^+/−^ MEF cells, and secondly as a measure of their ability to repair the damage^40^. Wild-type control and *Tcof1*^+/−^ MEF cells were treated with the oxidant H_2_O_2_ for 30 min, after which fragmented DNAs were visualized by SYBR Green at 0, 60 and 90 min following release from oxidation. In control wild-type MEF cells, DNA damage was completely repaired within 60 min of washing out H_2_O_2_. In contrast, in *Tcof1*^+/−^ MEF cells, fragmented DNAs were present for considerably longer, even >90 min post H_2_O_2_ exposure (Fig. 5c,d). Collectively these observations indicate that *Tcof1*/Treacle is required for proper DNA damage repair in response to oxidative stress during embryogenesis. Consequently, the accumulation of DNA damage and subsequent tissue-specific neuroepithelial apoptosis observed in *Tcof1*^+/−^ embryos influences the pathogenesis of TCS characteristic craniofacial anomalies.

### Antioxidants ameliorate craniofacial defects in *Tcof1* ^+/−^ mice

**Figure 6:**
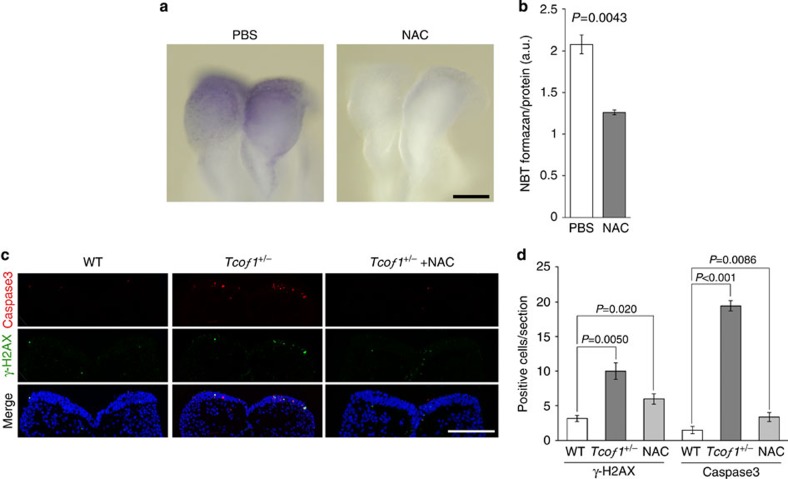
Prevention of DNA damage and subsequent apoptosis through the pharmacological suppression of endogenous ROS. (**a**) Pregnant dams were treated with the NAC through intraperitoneal injection at E8.5. After 1 h, endogenous oxidation was visualized by NBT staining. Scale bar, 100 μm. (**b**) Endogenous ROS levels were quantified by photometric assay of NBT formazan. The amount of NBT formazan in control (PBS; white bars) and NAC-treated embryos (grey bars) are shown as mean± s.e. of three embryos. Statistical differences were assessed with Student's *t*-test, and *P* values were shown. (**c**) Pregnant dams were treated with the NAC through intraperitoneal injection from E5.5 to E8.5. DNA damaged and apoptotic cells were detected by immunostaining of γ-H2AX and cleaved Caspase 3 using cryosections of anterior neural plate (fore–midbrain level). Scale bar, 100 μm. (**d**) The number of γ-H2AX-positive and cleaved Caspase 3-positive cells on a section prepared from the anterior neural plate (fore–midbrain level) of wild-type (white bars), *Tcof1* mutant (grey bars) and NAC-treated *Tcof1* mutant embryos (light grey bars) are shown as means±s.e. of 12 sections prepared from four embryos. Statistical differences were assessed with Student's *t*-test, and *P* values were shown.

Our results indicate a relationship between oxidative stress, DNA damage and the apoptotic elimination of neuroepithelial cells in the pathogenesis of TCS. This observation raised the intriguing possibility that reducing the levels of endogenous oxidative stress through antioxidant supplementation might be able to ameliorate the pathogenesis of craniofacial abnormalities in *Tcof1*^+/−^ embryos. As a proof of principle, we cultured control wild-type E8.5 whole mouse embryos in the presence of N-acetyl-cystein (NAC), which is a strong antioxidant, and observed that NAC can prevent formazan formation (Fig. 5a; +NAC) thus demonstrating the ability of NAC to scavenge ROS *in vitro* in cultured mouse embryos. We next evaluated the efficiency of NAC to scavenge ROS via intraperitoneal injection of pregnant females. A single 150 mg kg^−1^ injection of NAC was sufficient to reduce formazan formation in wild-type embryos (Fig. 6a,b) demonstrating the efficacy of NAC to scavenge ROS *in utero*. Therefore we treated *Tcof1*^+/−^ embryos *in utero* with NAC (150 mg kg^−1^) via daily intraperitoneal injection of pregnant females from E5.5 to E8.5 with control litters being administered a similar regime using phosphate-buffered saline (PBS). In control wild-type embryos, there is little evidence for the presence of DNA damage in the neuroepithelium, nor neuroepithelial cell apoptosis. In contrast, *Tcof1*^+/−^ embryos exhibit high levels of DNA damage (Supplementary Fig. 4a,b and Fig. 6c,d; γH2AX^+^) in concert with high levels of neuroepithelial apoptosis (Fig. 6c,d; Caspase 3^+^). In *Tcof1*^+/−^ embryos treated with NAC, the number and size of individual DNA damage-induced foci were noticeably reduced and consistent with this, neuroepithelial apoptosis was considerably diminished (Fig. 6c,d; γ−H2AX^+^ and Caspase 3^+^). We further evaluated the reduction in DNA damage post-NAC treatment via immunostaining for the DNA damage marker P-Chk2, and also p53 which is phosphorylated and stabilized by P-Chk2. Both P-Chk2 and p53 activity were significantly reduced in NAC-treated *Tcof1*^+/−^ embryos compared with untreated *Tcof1*^+/−^ embryos. In fact the activity was reduced to levels similar to that observed in wild-type controls (Supplementary Fig. 9).

**Figure 7:**
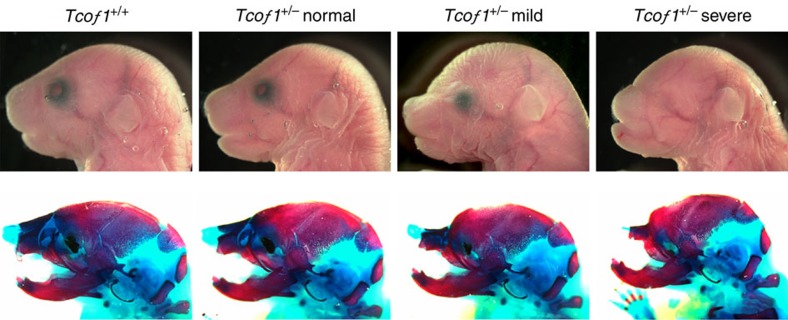
Pharmacological prevention of craniofacial malformation. *Tcof1*^+/−^ embryos were treated with (NAC) or without (control; PBS) antioxidant *in utero* from E5.5 to E10.5 (short) or to E17.5 (long) via daily intraperitoneal injection of pregnant mothers. Embryos were collected at E18.5–19.0 and subjected to skeletal analysis. Both short- and long-term antioxidant supplementation repressed the severe phenotype and ameliorated the presence of craniofacial anomalies in approximately 30% of treated embryos.

We previously showed that p53 inhibition was sufficient to suppress neuroepithelial cell death in *Tcof1*^+/−^ embryos and prevent the pathogenesis of craniofacial anomalies^7^. Therefore, we hypothesized that if oxidative stress-induced DNA damage was a potential trigger for p53 activation in *Tcof1*^+/−^ embryos, then dietary antioxidant supplementation may also be capable of ameliorating or even preventing cranioskeletal anomalies in mouse models of TCS. *Tcof1*^+/−^ embryos were treated *in utero,* either short-term from E5.5 to E10.5 or long term from E5.5 to E17.5, via daily intraperitoneal injection of pregnant mothers with NAC (150 mg kg^−1^) (Fig. 7; Table 1). To evaluate the efficacy of NAC treatment, cranioskeletal phenotypes of E18.5–19.0 embryos were categorized into three classes; (1) severe—defined by a domed head together with substantial hypoplasia of the cranial vault, nasal bone, premaxilla and maxilla bones. These embryos also typically exhibited anophthalmia; (2) mild—defined by moderate hypoplasia of the cranial vault as well as reductions in the nasal, premaxilla and maxilla bones. These embryos also frequently exhibited microphthalmia; (3) normal—indicative of an appearance indistinguishable from wild type (Fig. 7).

In untreated litters, *Tcof1*^+/−^ embryos predominantly presented with the severe phenotype (82.4%; Fig. 7; Table 1). The remaining 17.6% of *Tcof1*^+/−^ embryos were classified as mild. On no occasion was a *Tcof1*^+/−^ embryo identified that could be classified as normal and identical to wild type. In contrast, short-term NAC antioxidant supplementation prevented the pathogenesis of craniofacial anomalies in 30.7% (4/13) of *Tcof1*^+/−^ embryos such that their gross appearance and alizarin red (bone)/alcian blue (cartilage) stained craniofacial skeleton appeared identical to their wild-type littermates (Fig. 7; Table 1). Furthermore, antioxidant supplementation increased the ratio of mild phenotypes from 17.6 to 38.5% (5/13) while concomitantly reducing the incidence of severe phenotypes from 82.4 to 30.7% (4/13). Similarly, long-term NAC antioxidant supplementation ameliorated the pathogenesis of craniofacial anomalies in 30.0% (6/20) of *Tcof1*^+/−^ embryos, and increased the ratio of mild phenotypes from 17.6 to 45.0% (9/20) while concomitantly reducing the incidence of severe phenotypes from 82.4 to 25.0% (5/20; Fig. 7; Table 1). The fully rescued *Tcof1*^+/−^ embryos were morphologically indistinguishable from their control wild-type littermates. The cranial vault, nasal, premaxilla and maxilla bones, as well as the eyes, all appeared normally formed (Fig. 7). Therefore NAC antioxidant treatment during the critical period of neural crest cell formation (E8.25–10.5) can dramatically ameliorate and even prevent the pathogenesis of severe craniofacial abnormalities in *Tcof1*^+/−^ embryos.

All *Tcof1*^+/−^ newborns on the DBA;C57BL/6 background die within 24 h of birth owing to an inability to feed or breathe properly. This is a consequence of their severe frontonasal hypoplasia which encompasses nasal septum agenesis and choanal agenesis together with cleft palate^6^. In a few instances, NAC-treated pregnancies proceeded to birth, and NAC-treated *Tcof1*^+/−^ pups exhibited prolonged survival until at least day 3. The craniofacial bones, cartilage and palate all appeared normally formed (Supplementary Fig. 10a,c; palate). However, coronal sections of the frontonasal region revealed that although the nasal cavities were largely restored, they remained incomplete (Supplementary Fig. 10b). The nostrils were smaller than those of wild-type littermates (Supplementary Fig. 10c; arrowheads) and consequently the nasal cavities became clogged with dander and other debris from their cages (Supplementary Fig. 10b; arrowheads). Nonetheless, our particular NAC antioxidant supplementation regime dramatically improved craniofacial bone, cartilage and palate formation and substantially restored nasal cavity and nostril development leading to an improvement in post-natal viability. Thus antioxidant supplementation can considerably ameliorate the pathogenesis of craniofacial anomalies in mouse models of TCS. Our expectation is that further refinement of the antioxidant treatment (for example, concentration, timing, or the use of a different antioxidant or combination thereof) could better restore nasal function and lead to prolonged post-natal viability.

## Discussion

Craniofacial anomalies account for approximately one-third of congenital birth defects. While post-natal treatment of complex craniofacial malformation syndromes through comprehensive, well co-ordinated surgery and rehabilitation can help ameliorate the problems, the results are often variable and rarely fully corrective. Therefore considerable effort needs to be invested in developing therapeutic avenues for prevention, but this can only come from a better understanding of the genetic and cellular mechanisms that regulate neural crest cell development in association with the etiology and pathogenesis of individual malformation syndromes. TCS is a rare disorder of craniofacial development, which is caused at the cellular level by a deficiency in the formation and survival of neural crest cells. *TCOF1*, the gene mutated in most cases of TCS, is known to be a critical regulator of ribosome biogenesis and it has been well established that deficient ribosome biogenesis can cause apoptosis via a nucleolar stress-induced, p53-dependent pathway^7^,^9^. Mechanistically, deficient ribosome biogenesis has been proposed to be responsible for the high levels of p53-mediated neuroepithelial cell death observed in *Tcof1*^+/−^ embryos, which could then account for the deficiencies in neural crest cell generation and proliferation that underscore the craniofacial malformations characteristic of TCS^6^. Consistent with this mechanism, inhibition of p53 suppresses apoptosis in *Tcof1*^+/−^ embryos and prevents the pathogenesis of craniofacial anomalies characteristic of TCS.

**Figure 8:**
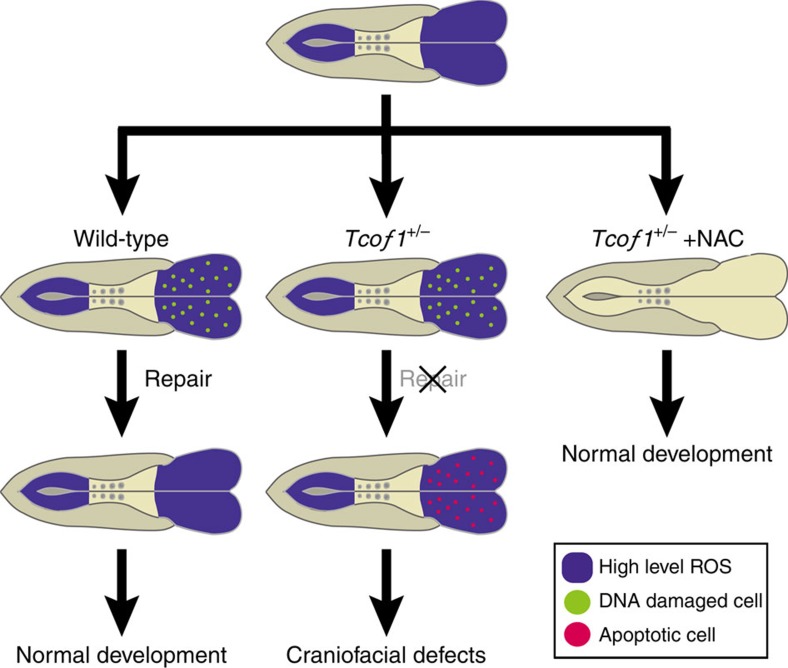
Summary of *Tcof1*/Treacle function in neuroepithelial survival and prevention of neuroepithelial apoptosis by NAC treatment. Our results uncovered that endogenous ROS accumulate in the anterior and posterior neuroepithelium of E8.5 embryos. Thus DNA damage response/repair machinery is required for prevention of oxidative DNA damage. In *Tcof1* mutant embryos, neuroepithelial cells undergo apoptosis due to the impairment of DNA damage repair function. In contrast, scavenge of ROS by antioxidant treatment represses apoptotic elimination of neuroepithelial cells, and rescue the craniofacial abnormalities in *Tcof1* mutants.

Currently more than 200 family specific mutations have been identified in the *TCOF1* gene in association with the etiology of TCS. Collectively, these mutations span the entire length of *TCOF1*, and are associated with a high degree of inter- and intra-familial variation in the severity of the craniofacial phenotype. However there is no clear evidence for a genotype–phenotype correlation^41^. Interestingly, we discovered that *TCOF1*/Treacle associates with the MRNM complex. Furthermore we found that neuroepithelial cells exist endogenously in a highly oxidative state and also that neuroepithelial cells are particularly sensitive to exogenous oxidative stress. Our results demonstrate that *Tcof1*/Treacle is required for DNA damage response/repair in the neuroepithelium, such that *Tcof1* loss-of-function leads to the apoptotic elimination of neuroepithelial cells (Summary, Fig. 8). Therefore we have uncovered a novel link between *Tcof1*/Treacle, oxidative stress-induced DNA damage, and neuroepithelial apoptosis in the pathogenesis of TCS

In support of our findings, two recent studies aimed at identifying interacting partners of NBS1 also found an association between NBS1 and TCOF1/Treacle^42^,^43^. Moreover, NBS1 was subsequently shown to bind to the N terminus of Treacle indicating that the interaction was direct. Furthermore, both NBS1 and Treacle were found at sites of DNA damage in nuclei and nucleoli in cultured cells *in vitro* following laser micro-irradiation^42^,^43^. We also observed the co-localization of Treacle in nucleoli in association with DNA damage. Thus, these studies independently confirm our identification of Treacle as part of the MRNM complex and its association with the DNA damage repair/response. In further agreement with our results, it was recently proposed that *TCOF1*/Treacle may play a role in oxidant defence^44^ such that *Tcof1* loss-of-function or haploinsufficiency could increase the sensitivity to oxidants. Our data illustrated the high sensitivity of neuroepithelial cells to oxidative stress. Considering that during pregnancy, each gestational environment subjects an embryo to distinct and varying levels of oxidative stress depending on the physiological conditions in the mother, this can help to explain the inter- and intra-familial variability in phenotypic severity observed in TCS. For example, maternal diabetes, smoking and alcohol consumption during pregnancy are all factors known to increase maternal ROS levels, which can be damaging to the genomic DNA of embryos^45^.

Many human syndromes are caused by deficiencies in DNA response/repair, and some such as in the case of mutations affecting the MRN complex are known to cause craniofacial anomalies^46^,^47^. For example, Nijmegen breakage syndrome (NBS), exhibits microcephaly as well as distinct facial features including a small lower jaw, and is caused by apoptosis of neuroepithelial cells^48^ in association with hypomorphic mutations in *NBS1*. Similarly, specific mutations in *MRE11* have also been shown to underlie craniofacial anomalies including a small jaw and chin, together with short palpebral fissures and microcephaly as part of the rare Ataxia Telangiectasia-like disorder^49^,^50^,^51^. Furthermore, neural tissue-specific disruption of *Nbs1* and *Mre11* via *Nestin-cre* conditional gene targeting, results in microcephaly as a consequence of massive neuroepithelial cell apoptosis^52^.

In addition, knockout of another DNA damage response/repair gene, *BRCA1* results in extensive neuroepithelial cell apoptosis such that the mouse embryos only survive until the early stages of craniofacial development^53^,^54^,^55^. Furthermore, an *Emx1-cre*-driven, neural-specific conditional deletion of *Brca1* results in neural progenitor apoptosis and subsequent brain defects^56^. Interestingly, Brca1-labelled DNA damage-induced foci were significantly reduced in *Tcof1*^+/−^ MEF cells, suggesting that *TCOF1*/Treacle may play an important role in the DNA damage response/repair through BRCA1 recruitment to and/or maintenance at sites of DNA damage. We were unable to detect any interaction between Treacle and BRCA1, which implies that Treacle may only interact with BRCA1 indirectly and through other partner(s). Consistent with this idea, work aimed at identifying binding partners of the C-terminal domain of BRCA1 suggested that Treacle may be indirectly linked to BRCA1 through ECT2 (ref. 57). Definitive proof however still requires further experimentation. Taken together, however, these observations strongly support our hypothesis that neural crest and brain tissues which are derived from neuroepithelial cells rely heavily on the DNA damage response and repair system for survival under conditions of high endogenous or exogenous oxidative stress.

In contrast to full knockout models of *Nbs1*, *Mre11 and Brca1*, which are early embryonic lethal^58^,^59^, *Tcof1*^+/−^ mice survive until birth. This facilitated our assessment of the importance of the DNA damage response/repair pathway in neuroepithelial survival during neural crest cell development and craniofacial morphogenesis in the pathogenesis of TCS.

While many DNA damage response/repair genes are ubiquitously expressed, *Tcof1* is expressed strongly in the neuroepithelium including neural progenitor and neural crest progenitor cells of E8.5 embryos^6^. Furthermore, DNA damage accumulation and cell death were detected specifically in the neuroepithelium of *Tcof1*^+/−^ embryos. Interestingly, our studies revealed that Treacle can form a complex with MRNM proteins in the absence of DNA damage, which implies that Treacle may exist in a poised form, ready to respond quickly to genotoxic stress-induced DNA damage when required. Thus *Tcof1*/Treacle activity in the neuroepithelium may be required for a rapid response to DNA damage induced by endogenous ROS for neuroepithelial cell survival during embryogenesis. Hence *Tcof1/*Treacle could function to protect neural progenitor and neural crest progenitor cells against endogenous oxidative stress-induced cell death during embryonic development. The complete molecular mechanism by which Treacle enhances the DNA damage response and repair pathway remains to be elucidated and further detailed investigations are needed to understand this mechanism at the molecular level. It is important to note though that Treacle was recently identified as a potential substrate for ATM and ATR, both of which are critical for activating γ-H2AX signalling in response to DNA damage^60^. This implies that Treacle phosphorylation by ATM or ATR may be necessary for its DNA damage response activity and this will be an important avenue to explore in the future.

These novel findings for *Tcof1/*Treacle function do not negate the idea that TCS is a ribosomopathy. In fact, the classification of TCS as a ribosomopathy is strengthened by the recent identification of causative autosomal recessive mutations in *POLR1C* and autosomal dominant mutations in *POLR1D* in association with TCS^61^. POLR1C and POLR1D are subunits of RNA polymerase I and III lending further support to the idea that TCS is a neurocristopathy arising from a defect in ribosome biogenesis. Rather, we view the role for *Tcof1*/Treacle in the DNA damage response as contributing factors to the variable severity of the phenotype of the disorder. In support of this idea, it is well established that DNA damage and ribosome biogenesis are inextricably linked as ribosome biogenesis is suppressed as a consequence of DNA damage^62^. In fact, DNA double strand breaks result in RNA Polymerase I transcriptional arrest. This response is very rapid as it is complete within 5 min of DNA damage^63^. Interestingly, P-ATM directly binds to and inhibits RNA polymerase I (ref. 63), and it is notable that P-ATM is considerably upregulated in *Tcof1*^+/−^ embryos. *Tcof1*/Treacle may therefore provide another direct link that co-ordinately regulates the DNA damage response with ribosome biogenesis, and in doing so helps to protect neural progenitor cells and neural crest cells from oxidative stress-induced damage, thus ensuring their proliferation and survival.

Apoptosis can be induced in response to deficiencies in ribosome biogenesis or as a consequence of DNA damage, both of which play a role in the pathogenesis of TCS. Consistent with the proposed novel role for *TCOF1*/Treacle, we demonstrated that antioxidant treatment could significantly ameliorate the pathogenesis of severe craniofacial malformations in *Tcof1*^+/−^ mice (Summary, Fig. 8). Thus the key to potentially preventing TCS lies in the suppression of apoptosis which helps to ensure the generation of sufficient neural crest cells to make a complete craniofacial skeleton. In this regard NAC antioxidant administration during the critical period of neural crest cell formation (E8.25–10.5) can suppress cell death in the neuroepithelium. In doing so, antioxidant treatments can dramatically ameliorate and even prevent the pathogenesis of severe craniofacial abnormalities in *Tcof1*^+/−^ embryos. Previously we discovered that genetic and pharmacological p53 inhibition could successfully prevent the pathogenesis of craniofacial anomalies in neurocristopathies such as TCS^7^, however this approach carries a substantial tumorigenesis risk. Thus, antioxidant supplementation and its inhibition of oxidative stress-induced DNA damage may be a more realistic and viable avenue for the clinical prevention of TCS rather than p53 inhibition.

## Methods

### Mice

All experiments were approved by the Institutional Animal Care and Use Committee of the Stowers Institute for Medical Research. *Tcof1*^+/−^ mice were maintained on a pure DBA background^64^. We bred DBA *Tcof1*^+/−^ mice and C57BL/6 wild-type mice to generate F1 progeny which are neonatal lethal and present with craniofacial anomalies that consistently model the severe form of Treacher Collins syndrome as previously described^6^,^7^. Mutant embryos were obtained by timed mating with the morning of the vaginal plug designated as embryonic day (E) 0.5. A minimum sample size of five individuals was used in each assay unless otherwise described.

### DNA constructs and siRNA analysis

The full-length mouse *Tcof1* cDNA was obtained from IMAGE clones (ID 6825735-2). A FLAG-tag sequence was fused in-frame to the N terminus of *Tcof1* by PCR. FLAG-tagged *Tcof1* full-length sequence was cloned into pcDNA5/FRT to generate FLAG-*Tcof1*-pcDNA5/FRT. Three kinds of Stealth siRNA (Life Technology; HSS110575, 110248 and 110249) and a custom siRNA (SMARTpool, Thermo Scientific) against *TCOF1* were purchased. Each siRNA was transfected into HeLa cells and knockdown efficiency was assessed by RT-PCR. Stealth siRNA HSS110575 was the most efficient as shown in Supplementary Fig. 5, and thus used in this study as siTCOF1. Control siRNA for Luciferase (GL2) was purchased from Thermo Scientific. siRNAs against *MDC1* (ref. 18) as previously described were synthesized by Thermo scientific.

### Cell culture and production of cell lines

HeLa, HEK293-T, 293-FRT and MEF cells were grown in DMEM containing 10% fetal bovine serum (FBS) and antibiotics. All cells were cultured at 37 °C in a humidified incubator with 5% CO_2_. Plasmid transfection was performed using LipofectAMINE 2000 (Invitrogen) following the manufacturer's protocol. For RNAi knockdown experiment, cells were transfected with siRNA using Lipofectamine RNAiMAX following the manufacturer's protocol after which cells were cultured for 48 h. To establish a stable cell line expressing FLAG-tagged Treacle, 293-FRT cells were transfected with FLAG-*Tcof1*-pcDNA5/FRT and pOG44 (Invitrogen) using LipofectAMINE 2000. Recombinants were selected and obtained via culture with hygromicin B (100 μg ml^−1^). Resistant cells were cloned and propagated and examined for their expression of Treacle. Cells stably expressing FLAG-Tcof1 was kept as Tcof1/293-FRT and used in this study. To prepare MEF cells, E11.5 *Tcof1*^+/−^ and wild-type littermates were collected and except for the head and internal organs, were minced by razor and incubated in 0.05% Trypsin–EDTA for 15 min at 37 °C. Dispersed cells were collected by centrifuge, resuspended in DMEM containing 10% FBS and seeded on 6 cm culture dishes.

### Immunoprecipitation and MudPIT analysis

For detection of interaction between endogenous Treacle or FLAG-Tcof1 and endogenous MRNM proteins, HEK293-T (for endogenous TCOF1 immunoprecipitation) or *Tcof1*/293-FRT and 293-FRT (for FLAG-Tcof1 immunoprecipitation) cells were lysed in lysis buffer 1 (50 mM Tris-HCl (pH 7.5), 120 mM NaCl, 0.5% NP-40, 1 mM EDTA and proteinase inhibitor cocktail (Nacalai tasque)). Whole-cell extracts were supplemented with 2 mM MgCl_2_ and benzonase (50 U ml^−1^) and incubated for 30 min at 4 °C. Lysates were centrifuged and the supernatant was incubated with anti-TCOF1 antibody (Atlas Antibodies) or agarose beads conjugated anti-FLAG antibody (Sigma) at 4 °C overnight. For immunoprecipitation of endogenous Treacle, Protein A-sepharose 4B (Sigma) was added to lysates, and lysates were further incubated for 3 h at 4 °C. Antibody–protein complex was then precipitated by centrifugation, and washed three times with lysis buffer 1. Immunoprecipitated proteins were dissolved in SDS sample buffer, and then subjected into SDS–polyacrylamide gel electrophoresis. Co-precipitation of MRNM proteins was detected by western blotting using following antibodies; anti-Mre11 (Cell Signaling, 1/1,000), anti-Rad50 (GeneTex, 1/100), anti-Nbs1 (Novus, 1/1,000), anti-MDC1 (Abcam, 1/2,000), anti-Treacle (Atlas antibodies, 1/1,000) and anti-FLAG (Sigma 1/5,000) antibody. Uncropped scans of critical representative western blots are presented in Supplementary Fig. 11. For MudPIT analysis, *Tcof1*/293-FRT and 293-FRT cells were cultured in 15 cm dishes. Cells were incubated in lysis buffer 2 (50 mM Tris-HCl (pH 7.5), 150 mM NaCl, 1% NP-40 and 5 mM EDTA) for 30 min on ice. Lysates were centrifuged and the supernatant was incubated with agarose beads conjugated anti-FLAG antibody (Sigma) at 4 °C overnight. The beads were then precipitated by centrifugation, and washed 5 times with lysis buffer. Immunoprecipitated proteins were eluted by FLAG peptide (200 μg ml^−1^ in lysis buffer) and then precipitated by TCA. Interacting proteins were identified using MudPIT as previously described^10^,^11^.

### Immunofluorescence staining of DNA damage-induced foci

Cells were grown on glass cover slips, irradiated X-ray at 10 Gy and allowed to recover at 37 °C for the indicated times. The accumulation of DNA damage proteins was detected by immunofluorescence as follows. Cells were incubated with NE buffer (20 mM HEPES (pH.7.9), 20 mM NaCl, 5 mM MgCl_2_, 1 mM DTT, 0.5% Triton X-100 and Protease Inhibitor Cocktail (Sigma)) for 20 min on ice and then fixed with 1% paraformaldehyde (PFA) for 10 min at room temperature. For immunofluorescence of Rad50 and Nbs1, cells were fixed with 100% methanol for 1 h at −20 °C, and then permeabilized with 100% acetone for 10 min at −20 °C. For immunofluorescence of phosphorylated ATM and Treacle, cells were fixed with 4% paraformaldehyde for 10 min then permeabilized with 0.5% TritonX-100 for 15 min at room temperature. After blocking with 3% bovine serum albumin or 10% goat serum in TBST (TBS+0.1% Tween20) for 30 min, cells were incubated overnight with antibodies to phosphorylated ATM at S1981 (P-ATM, Rockland, dilution 1/1,000), γ-H2AX monoclonal (Milipore, dilution 1/400), γ-H2AX polyclonal (Cell Signaling, 1/200), Rad50 (GeneTex, 1/1,000), Nbs1 (Novus, 1/500), MDC1 (Abcam, dilution 1/800) 53BP1 (Cell Signaling, dilution 1/500), BRCA1 (Santa Cruz, dilution 1/100), Conjugated ubiquitin (FK2, Stressgene, 1/1,000), RAP80 (Bethyl, 1/5,000) and Treacle (054, 1/200). Cells were washed twice with TBST and incubated with appropriate secondary antibodies conjugated with Alexa 488 or 546 (Invitrogen) for 1 h at room temperature. Nuclei were stained with DAPI (4,6-diamidino-2-phenylindole). Fluorescence microscopy was performed on a LSM5 PASCAL confocal microscope (Carl Zeiss) using 40 × and 63 × objective lenses. Confocal optical slices were collected and maximum-intensity projections of 0.3 μm stacks were made with Zeiss LSM5 software. For validation of co-localization of DNA damage-induced foci, signal intensity of each channel along a line was measured, and intensity plots was generated by the Plot Profile function of ImageJ software.

### Alkaline comet assay

*Tcof1*^+/−^ and wild-type MEF cells were treated with100 μM H_2_O_2_ for 30 min at 4 °C. Cells were allowed to recover at 37 °C for 0, 60 and 90 min after which an alkaline comet assay was performed as previously described^65^. Comet images stained with SYBRGreen (Invitrogen, 1 μg ml^−1^) were captured using LSM5 PASCAL confocal microscope (Carl Zeiss). The extent of DNA damage was evaluated in terms of Tail moment (Tail length times Tail DNA%). Tail moment was determined using OpenComet plugin for ImageJ^66^.

### G2/M checkpoint assay

HeLa cells were transfected with siGL2 (control), siTCOF1 and siRNAs for *RAP80* and *BRCA1*. After 24 h of culture, cells were irradiated at 3 Gy. One hour post irradiation, transfected cells were fixed with 70% ethanol and stained with anti-phospho histone H3 antibody, followed by incubation with fluorescent secondary antibody. Stained cells were incubated with propidium iodide and analysed by flow cytometry to estimate the percentage of cells in each cell cycle.

### Detection of endogenous oxidation

E8.5 embryos were dissected and cultured in a whole-embryo culture chamber (BTC Engineering, Cambridge, UK) for 45 min in Opti-MEM (Invitrogen) containing 1% FBS and 250 μg ml^−1^ of NBT in a 5% O_2_; 5% CO_2_; 90% N_2_ atmosphere. Embryos were washed twice with PBS and fixed with 1% paraformaldehyde for 1 h at 4 °C. For quantification of oxidation levels, formazan precipitates were dissolved by addition of 84 μl of 2 M KOH and 72 μl of DMSO immediately after washing with PBS. The amount of reduced NBT was quantified by determination of absorbance at 630 nm and normalized for total protein. E8.5 embryos were also dissected and incubated with 250 μM of H_2_-DCFDA in Opti-MEM+1% FBS for 1 h at 37 °C in a 5% O_2_; 5% CO_2_; 90% N_2_ atmosphere. Embryos were briefly washed three times with HBSS, and then incubated with HBSS for 30 min at 37 °C with rotation. After washing with HBSS, fluorescence was detected by whole-mount imaging.

### Whole-embryo culture

Whole-embryo culture was performed as previously described^6^,^67^,^68^. Briefly E8.0 embryos were dissected and cultured in a 5% O_2_; 5% CO_2_; 90% N_2_, 37 °C atmosphere for 12 h in DR50 (50% rat serum; 50% DMEM/F-12), 2% glucose in the presence or absence of 3-NP(Sigma). Embryos were fixed with 1% paraformaldehyde for 16 h at 4 °C and then analysed for the induction of apoptosis by TUNEL staining using an In Situ Cell Death Detection Kit Fluorescein (Roche).

### Immunohistochemistry

Mouse tissues were fixed with 1% paraformaldehyde in PBS for 16 h at 4 °C and cryopreserved in 20% sucrose in PBS. Tissues were embedded in OCT and stored at −80 °C until use. Cryostat sections were cut at 10 μm and adhered to glass slides. Sections were washed with TBST, then incubated with 3% bovine serum albumin in TBST for 30 min at room temperature. Sections were incubated at 4 °C overnight with antibodies to γ-H2AX (Millipore, 1/200), P-Chk2 at T68 (Cell Signaling, 1/200), cleaved Caspase 3 (Cell Signaling 1/200) and p53 (Cell Signaling 1/100). Sections were washed three times with TBST for 10 min and then incubated with appropriate secondary antibodies conjugated with Alexa 488 or 546 (Invitrogen) for 1 h at room temperature. Nuclei were stained with DAPI and the sections were also processed for the detection of apoptosis via TUNEL staining. Fluorescence microscopy was performed on a LSM5 PASCAL confocal microscope (Carl Zeiss) using 10 × , 20 × , 40 × and 63 × objective lenses. Confocal optical slices were collected and maximum-intensity projection stacks were made with Zeiss LSM5 software.

### Western blot analysis

Cultured cell lines were lysed in lysis buffer 1 (50 mM Tris-HCl (pH 7.5), 120 mM NaCl, 0.5% NP-40, 1 mM EDTA, proteinase inhibitor cocktail (Nacalai tasque)). Whole-cell extracts were supplemented with 2 mM MgCl_2_ and benzonase (50 U ml^−1^) and incubated for 30 min at 4 °C. Lysates were centrifuged and the supernatant was collected. Proteins were detected by SDS–polyacrylamide gel electrophoresis followed by western blotting. P-ATM (S1981) and γ-H2AX were analysed in whole-embryo lysates extracted from individual E8.5 embryos, Treacle was analysed using HeLa cell lysates by western blotting. α-Tubulin was used as an internal control. We analysed each band by densitometry with ImageJ software normalized against α-tubulin value to quantify the relative levels of P-ATM and γ-H2AX proteins in wild-type and *Tcof1* mutant embryos. For Treacle in HeLa cells, qualify the relative levels of Treacle proteins in non-damaged and damaged cells with 10 Gy X-ray irradiation.

### Pharmacological inhibition of oxidative stress

NAC (150 mg kg^−1^) was injected intraperitoneally into pregnant C57BL/6 wild-type female mice that had been bred to DBA *Tcof1*^+/−^ male mice. Injections were performed as follows; once at E8.5 (Fig. 6a), twice daily from E5.5 to E8.5 (Fig. 6c), twice daily from E5.5 to E10.5 (Fig. 7, short), twice daily from E5.5 to E10.5 followed by once a day from E11.5 to E17.5 (Fig. 7, long, Supplementary Fig. 10). Embryos were collected at 1 h after (Fig. 6a), E8.5 (Fig. 6c), E18.5–E19.0 (Fig. 7) and post-natally day 3 (P3) pups (Supplementary Fig. 10). For histological analysis, embryos and pups were processed for skeletal staining. To evaluate the efficacy of NAC supplementation, cranioskeletal phenotypes were categorized into three classes; (1) severe—defined by a domed head together with substantial hypoplasia of the cranial vault, nasal bone, premaxilla and maxilla bones. These embryos also typically exhibited anophthalmia; (2) mild—defined by moderate hypoplasia of the cranial vault as well as reductions in the nasal, premaxilla and maxilla bones. These embryos also frequently exhibited microphthalmia; (3) normal—indicative of an appearance indistinguishable from wild type.

### Skeletal analysis

E18.5–E19.0 embryos and P3 pups were fixed in 99% ethanol and the bone and cartilage with stained with alizarin red and alcian blue respectively as previously described^69^.

### Statistics

For statistical analysis, two-tailed Student's *t*-tests were used to determine *P* values. *P* values of <0.01 were considered significant.

## Additional information

**How to cite this article**: Sakai, D. *et al.* Prevention of Treacher Collins syndrome craniofacial anomalies in mouse models via maternal antioxidant supplementation. *Nat. Commun.* 7:10328 doi: 10.1038/ncomms10328 (2016).

## Supplementary Material

Supplementary Information Supplementary Figures 1-11

## Figures and Tables

**Table 1:** Craniofacial phenotype with or without NAC treatment.

**Phenotype**	***Tco**f**1***^***+/+***^	***Tco**f**1***^+/−^		
	**Normal** **(%)**	**Normal**	**Mild**	**Severe**
Control	21 (100)	0 (0)	3 (17.6)	14 (82.4)
NAC (short)	13 (100)	4 (30.7)	5 (38.5)	4 (30.7)
NAC (long)	13 (100)	6 (30.0)	9 (45.0)	5 (25.0)
