# Supplementary material for: Prevention of Treacher Collins syndrome craniofacial anomalies in mouse models via maternal antioxidant supplementation
Source: Nat Commun. 2016 Jan 21;7:10328. doi: 10.1038/ncomms10328 (PMC4735750; doi:10.1038/ncomms10328)
Supplement: Supplementary Information — Supplementary Figures 1-11 [file ncomms10328-s1.pdf]

a

	Tcof1 dNSFA (x1000)	control dNSFA (x1000)
Mre11	1.0	0
Rad50	1.9	0
Nbs1	0.5	0
MDC1	0.09	0

b

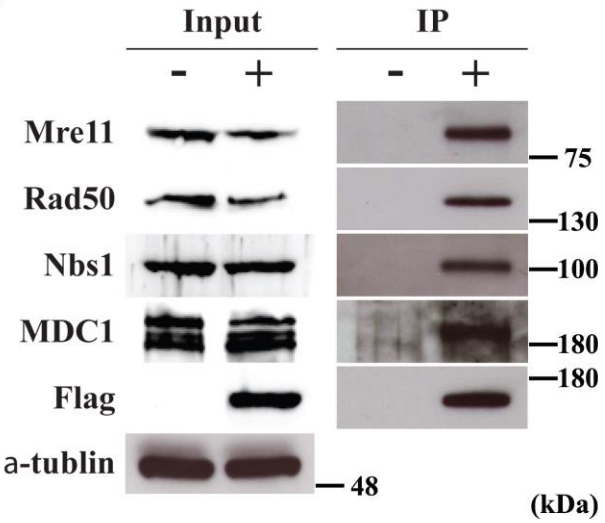

Supplementary Fig. 1. Treacle interacts with Mre11-Rad50-Nbs1-MDC1 complex. (a) Immunoprecipitated proteins were identified by MudPIT mass spectrometry. The normalized spectral abundance factor (NSAF) for Mre11, Rad50, Nbs1 and MDC1 in stable cell line expressing FLAG-tagged Treacle (Tcof1) or parental cell line (control) is shown. (b) Interactions between FLAG-tagged Treacle and endogenous Mre11, Rad50, Nbs1 and MDC1 were detected by immunoprecipitation (IP). Stable cell lines expressing FLAG-tagged Treacle (+) or parental cell line (-) were lysed, and then IP was performed using anti-FLAG antibody conjugated beads. Endogenous proteins in the IPed fraction were detected using antibodies to the proteins indicated on the left side.

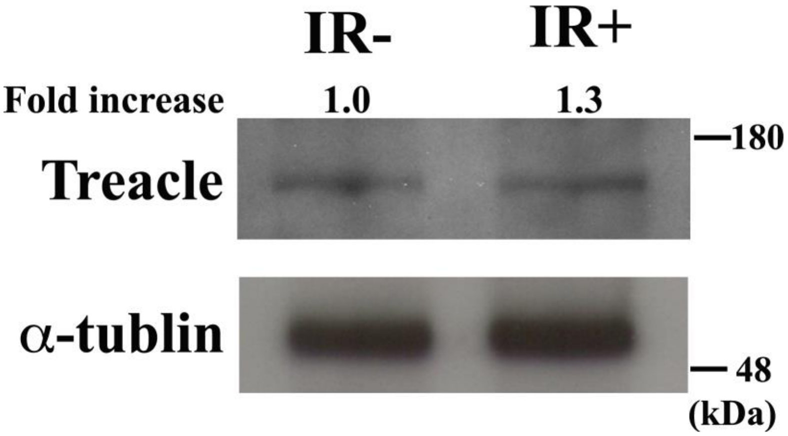

Supplementary Fig. 2. Treacle expression level is not altered upon irradiation. HeLa cells were treated (IR+) or untreated (IR-) with 10 Gy of X-ray. The amount of Treacle protein was quantified by western blotting using an anti-Treacle antibody. Signals were normalized to α-tubulin signals. Fold increase of relative values are presented.

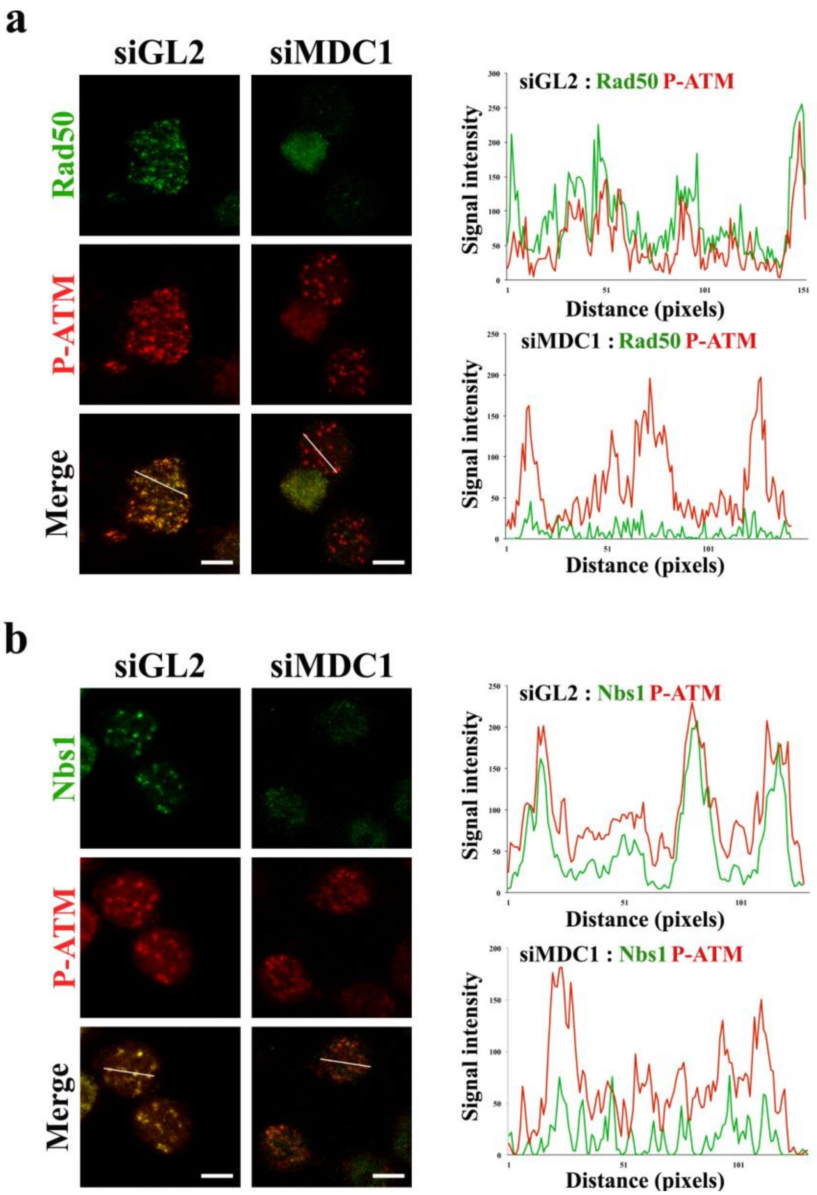

Supplementary Fig. 3. Silencing of MDC1 prevents DNA damage induced foci of Rad50 and Nbs1. Immunofluorescence images of MDC1 knockdown cells. HeLa cells transfected with control (siGL2) and siMDC1 were treated with radiomimetic drug, bleomycin, and then immunostained with antibodies to Rad50 (a), Nbs1 (b) and P-ATM. Scale bar, 5 μm. Plot profiles show signal intensities along an oblique line quantified by ImageJ (3 independent experiments, 1 representative image shown).

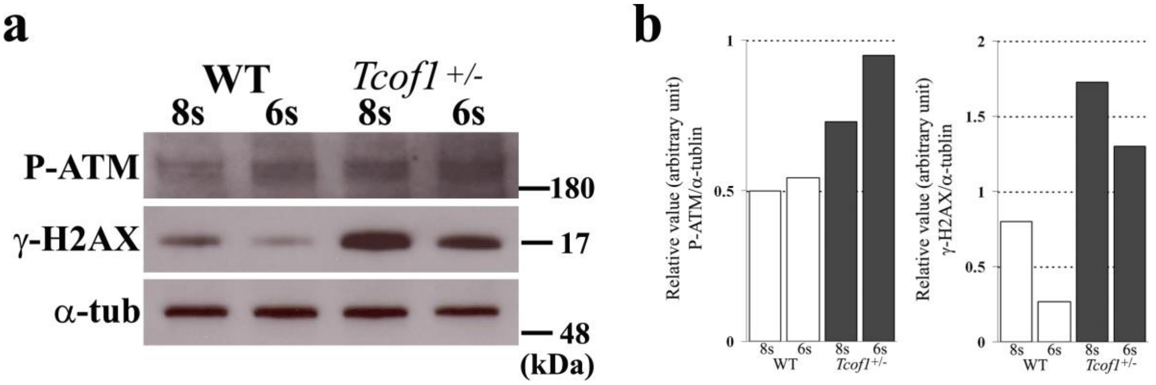

Supplementary Fig. 4. DNA damage accumulates in the neuroepithelium of *Tcof1*$^{+/-}$ embryos. (a) Accumulation of P-ATM and γ-H2AX in E8.5 Wild-type (WT) and *Tcof1*$^{+/-}$ embryo was quantified by western blotting using anti-P-ATM and anti-γ-H2AX antibodies. (b) Signals were normalized to α-tubulin signals and relative values are presented as bar graphs. P-ATM levels are 1.7- and 1.5-fold higher, γ-H2AX levels are and 4.8- and 2.2-fold higher at 6 and 8 somite stages, respectively in *Tcof1*$^{+/-}$ embryos (gray bar) compared to wild-type (WT; white bar).

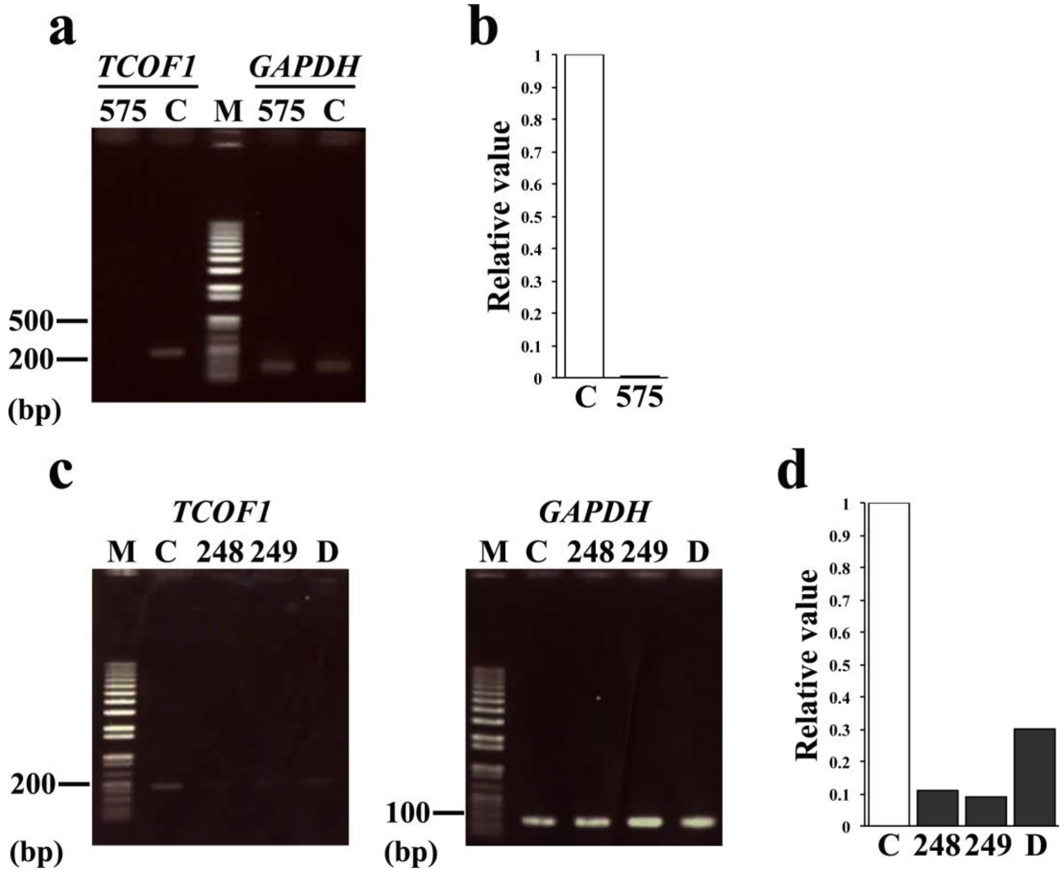

Supplementary Fig. 5. Comparison of knockdown efficacy of siRNAs against *TCOF1*.

(a, c) RT-PCR analysis of efficacy of *TCOF1* knockdown in HeLa cells 48 hours after control siGL2 (C), Invitrogen's Stealth siRNA against *TCOF1* HSS110575 (575), HSS110248 (248), HSS110249 (249) or custom siRNA against *TCOF1* synthesized by Dharmacon (D) transfection. *GAPDH* is used as internal control. The 1 kb DNA size marker is shown (M). (b, d) Bar graphs show relative values of *TCOF1* expression normalized with *GAPDH* expression in siRNA transfected cells. Expression values of *TCOF1* siRNA transfected cells were normalized to that of control siRNA transfected cells in order to represent expression ratios.

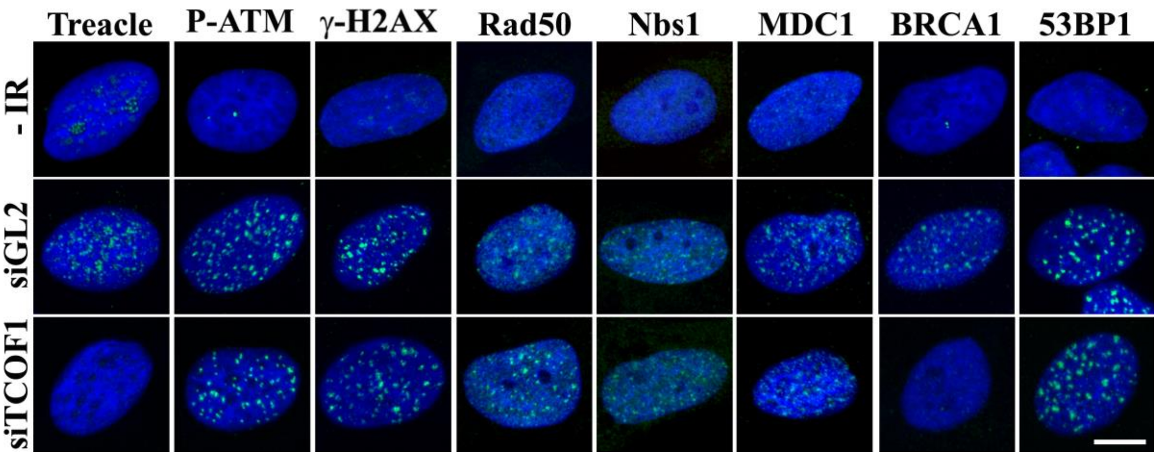

Supplementary Fig. 6. Loss of *TCOF1* leads to mislocalization of BRCA1. Immunofluorescence images of *TCOF1* knockdown cells. HeLa cells transfected with control (siGL2) and *Tcof1* siRNA (siTcof1) were immunostained with the antibodies indicated 1 hour after X-ray irradiation. Only BRCA1 foci were significantly decreased by *Tcof1* knockdown. Cells without irradiation (IR-) were used as a negative control. Scale bar, 5 μm.

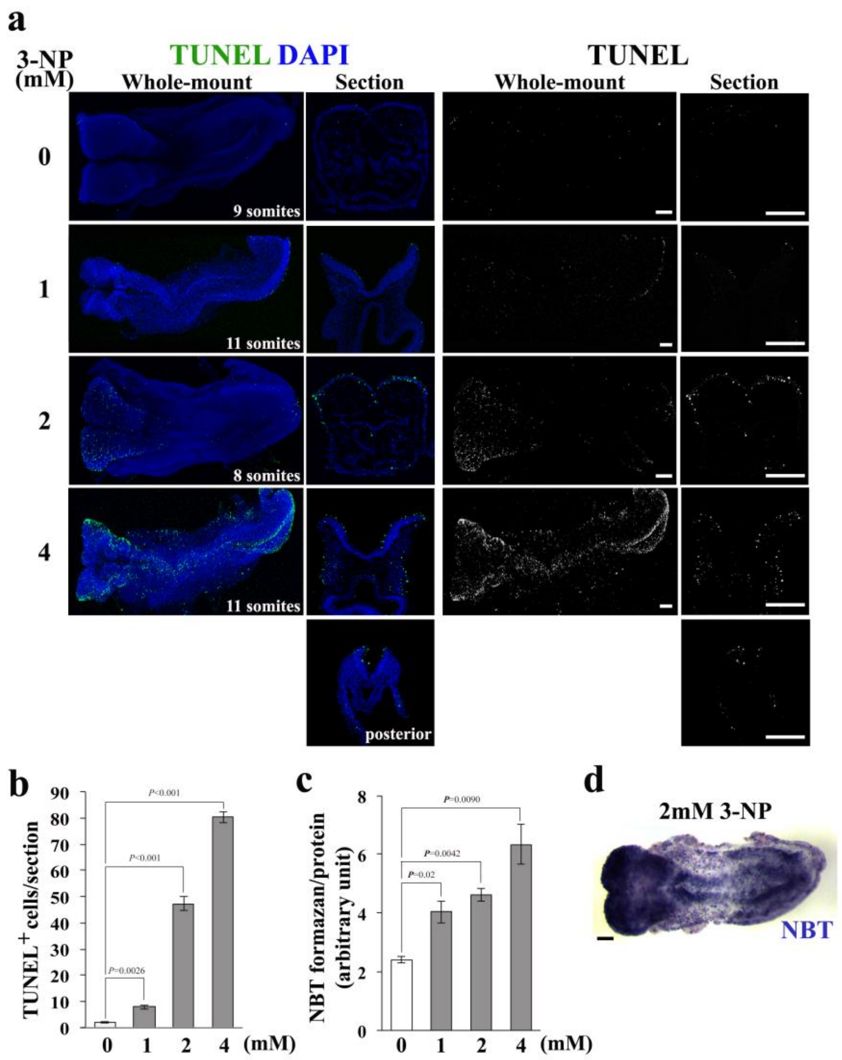

Supplementary Fig. 7. High sensitivity of the neuroepithelium to oxidative stress. (a) Apoptosis induced by 3-NP treatment was analyzed by whole-mount and section TUNEL staining. Scale bar, 200 μm. (b) The number of apoptotic cells in the anterior neural plate in 3-NP treated (gray bars) and non-treated (white bar) embryos are shown as means ± s.e. of 15 sections from 3 embryos. Statistical differences were assessed with Student's *t*-test, and P values were shown. (c) The amount of NBT formazan in non-treated (white bars) and 3-NP treated embryos (gray bars) are shown as means ± s.e. of 4 embryos. Statistical differences were assessed with Student's *t*-test, and P values were shown. (d) Oxidative state in 2mM 3-NP treated embryo was analyzed by NBT staining. ROS levels were increased throughout the embryo by 3-NP treatment. Scale bar, 200 μm.

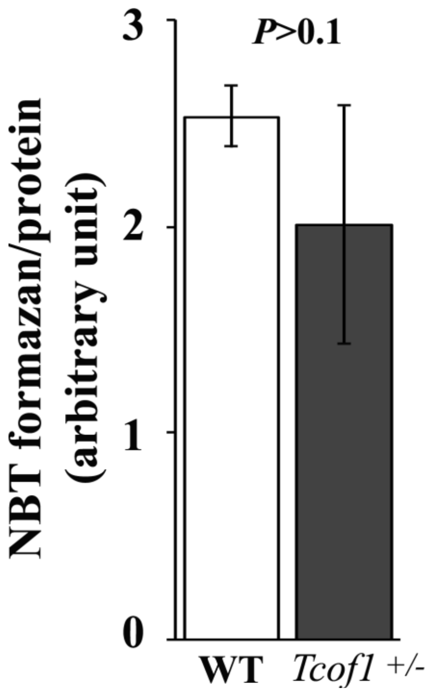

Supplementary Fig. 8. Endogenous ROS levels are not increased in *Tcof1*$^{+/-}$ embryos. Endogenous ROS levels were quantified by photometric assay of NBT formazan. The amount of NBT formazan in wild-type (WT; white bars) and *Tcof1*$^{+/-}$ embryos (gray bars) are shown as means ± s.e. of 6 embryos. Statistical differences were assessed with Student's *t*-test, and P values were shown.

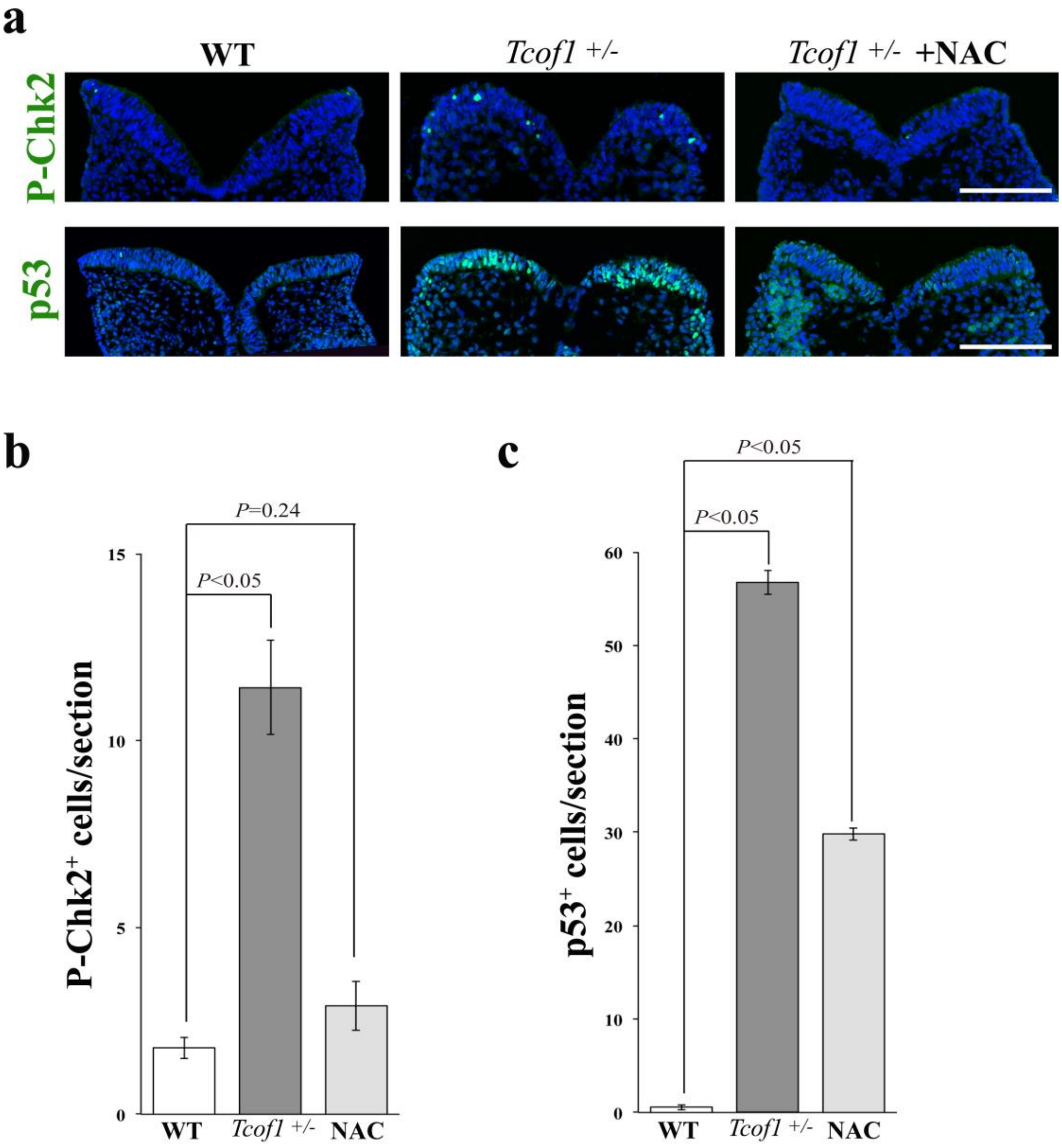

Supplementary Fig. 9. Prevention of DNA damage and subsequent activation of p53 through the pharmacological suppression of endogenous ROS. (a) Pregnant dams were treated with the NAC through intraperitoneal injection from E5.5 to E8.5. DNA damaged and apoptotic cells were detected by immunostaining of P-Chk2 and p53 using cryosections of anterior neural plate (fore-midbrain level). Scale bar, 100 μm. (b) The number of P-Chk2-positive and p53-positive cells on a section prepared from the anterior neural plate (fore-midbrain level) of wild-type (white bars), *Tcof1* mutant (gray bars) and NAC-treated *Tcof1* mutant embryos (light gray bars) are shown as means ± s.e. of fifteen sections prepared from 3 embryos. Statistical differences were assessed with Student's *t*-test, and *p*-values were shown.

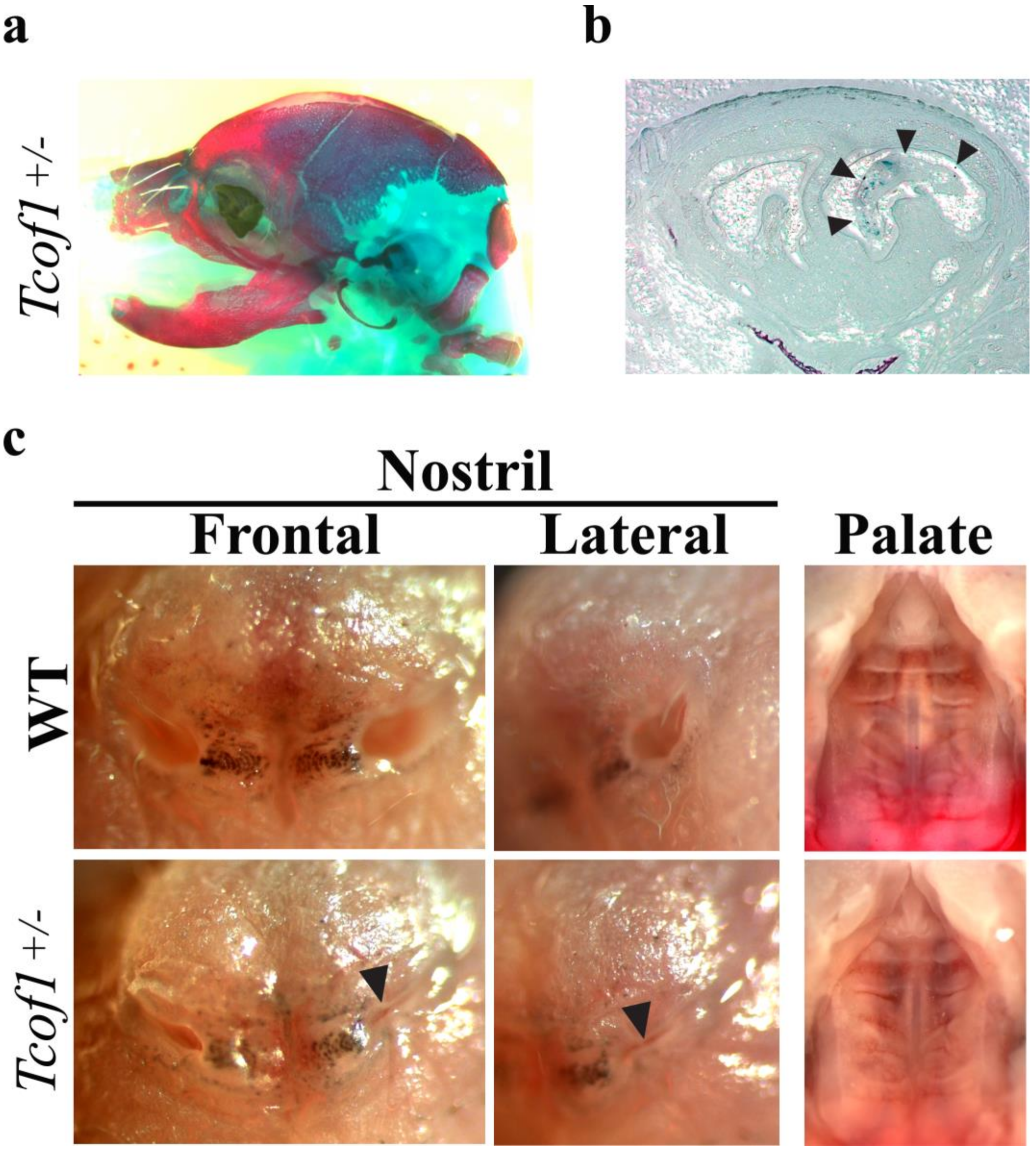

Supplementary Fig. 10. Rescue in nasal cavity and nostril formation. (a) Craniofacial skeletons of postnatal day 3 NAC-treated *Tcof1*$^{+/-}$ pup. (b) Coronal section of (a) at the frontonasal level. Arrowheads indicate clogs in nasal cavity. (c) Comparison of the palate and nostrils between NAC-treated wild-type (WT) and *Tcof1*$^{+/-}$ pup. Palate formation was rescued and while nostril formation was substantially restored, it remained incomplete.

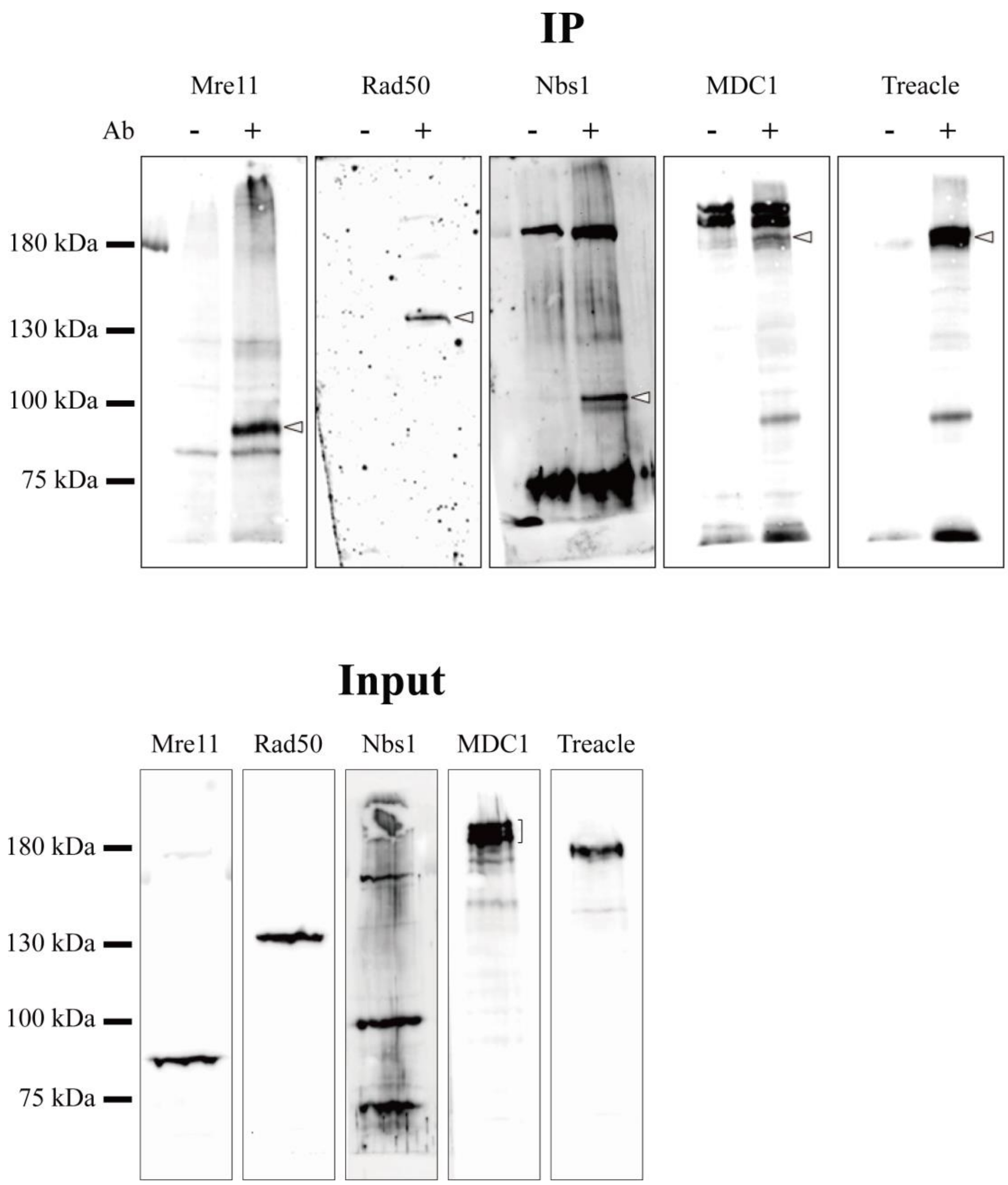

Supplementary Fig. 11. Uncropped scans of critical representative western blots in Fig, 1 highlighting the association between Treacle and MRE11, Rad50, Nbs1 and MDC1 which constitute the MRNM complex.
